# A protocol for investigating long-term social discrimination memory: Evidence in female and male Long Evans rats

**DOI:** 10.1371/journal.pone.0311920

**Published:** 2024-11-21

**Authors:** Fardad Pirri, Francine F. Burke, Cheryl M. McCormick

**Affiliations:** 1 Department of Biological Sciences, Brock University, St. Catharines, Canada; 2 Department of Psychology, Brock University, St. Catharines, Canada; Kyoto University Graduate School of Informatics: Kyoto Daigaku Daigakuin Johogaku Kenkyuka, JAPAN

## Abstract

Social discrimination, the investigation of a novel peer more so than a familiar peer, is used as a measure of social memory. There is much less research on long-term social memory than short-term social memory, and no long-term social memory research in female rats. The majority of long-term social discrimination research has relied on long familiarization session of an hour or more and involved juveniles as the stimulus peers. Here we show that a 30-minute familiarization session is sufficient to produce social discrimination 24 h later in both male and female rats and allows for measurement of social approach. Other methodological considerations are described, such as: that age- and sex-matched stimulus peers can be used across a wider range of ages than the use of juveniles; evidence that a familiar peer in a novel location attenuates social discrimination; that the first 10 minutes of the social approach reliably shows a preference for the social peer over an object whereas the 30-minute session does not; and that 10-minute discrimination sessions are preferable to 5-minute sessions. The research satisfies the goal of obtaining an efficient procedure to investigate both the possibility of enhancing or diminishing social approach and social memory with experimental manipulations in both sexes.

## Introduction

The ability to remember and differentiate among conspecifics is a fundamental feature of social behaviour. In laboratory settings, social memory was first studied in Long Evans rats in 1976 [1]. After a 10-minute familiarization session to the odour of either a juvenile or adult male and then a two-minute inter-session interval, when given the choice between two odours, male rats would prefer to investigate the novel rather than the familiar odour. Such a preference for novelty is the basis of many memory tests used in rodents, such as the object recognition memory test and the spatial location memory test [2, 3].

Although there are several types of social memory tests (for descriptions, see [4]), our focus is social memory tests that involve a familiarization session in which a rat investigates a conspecific stimulus rat (typically separated from the test rat in an enclosure) for a set amount of time and after an interval of time, the test rat has the opportunity to investigate either the now familiar conspecific or a novel conspecific. Such tests are referred to as social discrimination tests, with a preference for investigating the novel versus the familiar rat considered evidence of a social memory. The advantage of such tests is that only two test sessions are required (familiarization session and test session), whereas other procedures such as habituation-dishabituation procedures require more sessions (rats reduce investigation to a social stimulus rat presented several times, and greater investigation occurs upon presentation of a new social stimulus rat) (see [5] for additional advantages of the social discrimination procedure). Further, the familiarization phase can be used as a test of social approach (a.k.a. social preference or sociability) by having the stimulus rat in one compartment and an object in the other, although many studies of social discrimination leave one compartment empty during familiarization (e.g., [6]).

The social discrimination test is used widely in investigations of rats and has led to much knowledge of the neural mechanisms involved in social memory. Nevertheless, there is no standard way to carry out the procedure, which may underlie some discrepancies in results in the literature. Some researchers have the familiarization session conducted in the home cage and the test session in a different arena [7], which is not feasible for pair-housed rats. Further, a requirement of single housing may add isolation stress as a factor in the results. Most of the social memory tests in rats have involved short-term memory, with familiarization and test sessions of two (e.g., [8]), four (e.g., [9]), and up to twenty minutes (e.g., [10]). Short-term social memory is no longer evident when inter-session intervals are longer than two hours, which may reflect inefficient retrieval rather than a lack of memory storage [11]. Less than two percent of studies of social memory, however, have investigated long-term social memory, despite evidence that the underlying mechanisms differ between short- and long-term social memory [4].

There are fewer studies of long-term social memory in rats than in mice, for which social memory at 24 hours or more is readily found [12]. There was evidence of long-term social discrimination for odours of Lister Hooded rats that were cage mates for 18 days after a separation of 48 hours, and not after 96 hours [13]. In male Wistar rats, a social recognition memory procedure that allowed for physical interaction between rats (as opposed to the more typical use of a barrier to separate rats), a familiarization session of two hours with an adult male stimulus rat resulted in less investigation 24 h later in rats presented with the familiar rat than in rats presented with a novel rat [14]. Long-term social memory of up to seven days was found in both male and female rats when a habituation-dishabituation paradigm involving retroactive facilitation (three 5 min familiarization sessions separated by 10 min, then after one or seven days another exposure to the same juvenile for 5 min, and then a session with a novel juvenile 30 min later) [15]. A disadvantage of the latter procedure, however, is the number of trials involved. There is one report that two minutes of interaction with a juvenile was sufficient to result in social memory that lasted 72 hours (and not after seven days) in a recognition memory procedure in male rats (strain not indicated) [8]. This study, however, is in marked contrast to the vast number of studies indicating that familiarization sessions of up to twenty minutes in length do not lead to social memory beyond two hours in male rats. In social discrimination procedures in rats, there are several reports that a one-hour familiarization session resulted in long-term social memory observed at 24 h (e.g., [7, 16–22]) and, surprisingly, one study found 10 minutes of familiarization sufficient to produce long-term memory 24 h and seven days later [23]. None of these latter studies involved female rats nor Long-Evans rats.

The aim of the present studies was to investigate whether long-term social memory would be evident in female and male Long Evans rats using a 30-minute social approach test as the familiarization session. A goal was to have a protocol that could be used to investigate both the possibility of enhancing or diminishing social memory with experimental manipulations in both sexes. In the first two experiments, two social approach tests separated by 24 h were used to investigate the stability of social preference, with the social discrimination test session occurring after another 24 h. Our purpose for a two-day protocol for the social approach test was to allow in future research for groups that underwent stereotaxic surgery to be matched after the first test day before any pharmacological treatment and subsequent testing on the second test day. Further, because social approach tests have typically involved sessions of 3 to 15 minutes in length (e.g., [24–26]), we investigated whether the social preference would decline over the session. Although social approach tests are often referred to as social interaction tests, we prefer the term social approach to distinguish the test from the original social interaction test of File and Hyde (1978) [27] that involves physical contact between conspecifics rather than contact through a barrier.

Although social memory studies typically involved juveniles as the stimulus rats [4], we opted for adult peers of the same sex. A practical aspect of using adult stimulus rats is that they can be used for a longer timeframe than the shorter timeframe of juvenility. In experiment 2 we adapted the protocol based on the results of experiment 1 to remove novel location of the stimulus rat as a factor. The availability in the colony of rats of two ages allowed us to investigate whether social memory for age-matched stimulus rats would be evident at both 70 and 150 days of age. Although rats are considered to reach adulthood by 60 days of age, there is ongoing brain development beyond that age. For example, in the prefrontal cortex, serotonin transporter densities increase until 90 days of age [28] and dopamine D2 receptor densities decrease until 100 days of age [29]. Thus, age differences in social behaviour may be evident within that span of adulthood. In experiment 3, we investigated social memory after a 4 h interval to investigate whether performance would be enhanced relative to the social memory observed in experiment 2. In addition, because some researchers use test sessions of five minutes [18, 30] whereas others use ten minutes [22, 23] for the memory session, we investigated in all three experiments whether scores from the first five minutes or for the total 10-minute session resulted in stronger social memory.

## Materials and methods

### Experiment 1: Do both male and female Long Evans rats show evidence of social memory when tested after a 24 h interval?

#### Animals

Adult rats in this experiment were offspring of rats originally obtained from Charles River (St. Constant, Quebec) and raised in the Brock University animal facility. The offspring were weaned at postnatal day (PND) 21 and housed in same-sex pairs under standard laboratory conditions and kept on a 12-h light–dark cycle (lights on at 12:00 AM) with free access to food and water. All experiments were conducted during the dark phase and were approved by the Brock University Animal Care and Use Committee, and all procedures were carried out in accordance with the Canadian Council on Animal Care guidelines. Adult female and male rats (*n* = 24 and *n* = 24, respectively; aged 70–80 days of age) were the test rats and 12 female and 12 male rats served as stimulus rats. Rats were not handled before any experimental treatment other than for regular cage maintenance and to be weighed weekly.

#### Behavioural tests

*Social approach test (SAT)*. The modified version of the SAT was based on our previous publications [24, 31]. The SAT was performed during the early to late-dark phase within 2 to 9 hours after lights off in two separate rooms equipped with dim red light. Previous studies had sessions conducted under dim (e.g., [20, 21]) or white illumination [8] and less commonly in a dark room [7], although lighting condition in the test room is not always reported. We opted for testing under red light to minimize anxiety and all tests were conducted in the dark phase of the light cycle.

One hour before the test, test and stimulus rats were transferred from the colony room in their cages while covered with a dark blanket, and then housed in separate rooms illuminated with dim red light. The test arena was a white open-top melamine arena (60 cm x 30 cm x 60 cm) separated from two external chambers (30 cm × 30 cm × 30 cm) by a perforated plastic mesh that included a central hole (2 cm in diameter). The rats were habituated to the arena for 10 minutes, and test days began the next day. On test day 1, the external chambers contained an object (a plastic toy of the same size as a rat) in one end and an unfamiliar social stimulus (a same-sex and same-age peer) in the other, and the rats had 30 minutes to explore the stimuli. On test day 2, rats were placed inside the arena to approach or avoid chambers that contained a new unfamiliar stimulus (same-sex and same-age peer) or an object for 30 minutes. The arena was cleaned with Peroxigard before each test session. The chamber containing the unfamiliar rat was counterbalanced across rats. Stimulus rats (i.e., those used as unfamiliar rats in the chambers) were habituated to the chamber 24 h before test day 1 and were given a food reward (Froot Loops®) after each test session. Stimulus rats were never used in more than three sessions, and each session was spaced 90 minutes apart, with the stimulus rat returning to its home cage in between sessions. Sessions were recorded with a digital camera mounted at the ceiling above the test arena, and later analyzed with EthoVision software (version x17, Noldus Information Technology). For video analysis, we defined the social zone and object zone as a distance of 20 cm (approximately the body length of an adult rat) from the perforated mesh on each side of the arena, and a rat was considered within a zone when the centre point of the rat was inside a zone. Time spent in social zone, time spent in object zone and distance travelled in the test arena were the measures obtained. A social preference score also was calculated ((time spent in social zone / (time spent in social + object zones) * 100).

*Long-term social discrimination memory test (LTSDMT)*. Our social discrimination procedure was based on the original design of Engelmann and colleagues [5] in rats and the modifications of Moy and colleagues [32] in mice. Briefly, test day 2 of the SAT was used as a familiarization trial. 24 h after test day 2 of the SAT, each rat was placed inside the same test arena that now had a familiar rat (the previously unfamiliar rat from test day 2) in one chamber and a novel rat (same-sex and same-age peer) in the other chamber, and the test rat had 10 minutes to explore the social stimuli. One hour before the test, test rats and stimulus rats were transferred from the colony room in their cages while covered with a dark blanket, and then housed in separate rooms illuminated with dim red light to prevent any interactions before the start of the test. The LTSDMT was performed during the mid to late-dark phase within 6 to 9 hours after lights off in two separate rooms equipped with dim red light. The arena was cleaned with Peroxigard before each test session. Sessions were recorded via a digital camera located above the test arena and were analyzed with EthoVision software (time spent in familiar and novel zones). A social novelty preference score also was calculated (time spent in novel zone / (time spent in familiar + novel zones) * 100).

#### Experimental design

For the SAT, the stimulus rat was placed in either the left or right chamber (counterbalanced) during test day 1. For the SAT on test day 2, a new stimulus rat was placed on the opposite chamber than that of test day 1. A new object was placed in the other chamber on both test days. For the LTSMT half the rats had access to the familiar peer located on the same side as test day 2 and a novel rat positioned on the opposite side, and the other half had the familiar peer located in the opposite chamber than that of test day 2 and the novel rat placed in the other chamber.

### Experiment 2: Is LTSDM evident using older adult stimulus and test rats?

#### Animals

Female rats (*n* = 20 tested at postnatal day (PND) 78, and *n* = 26 tested at PND 150 days of age) were obtained from Charles River (St. Constant, Quebec) and housed in same-sex pairs in the Brock University vivarium under standard laboratory conditions and kept on a 12 h light–dark cycle (lights on at 12:00 AM) with free access to food and water. Rats were not handled before any experimental treatment other than for regular cage maintenance and to be weighed weekly.

#### Experimental design

The SAT and LTSDMT were conducted as in Experiment 1 with 28 test rats (12 rats 70 days of age and 16 rats 150 days of age) and 18 stimulus rats (8 rats 70 days of age and 10 rats 150 days of age). The only modification to test procedures was that stimulus rats were always placed in the same chamber (counter-balanced across rats) in all three test days, with an object in the other chamber on test days 1 and 2 and an unfamiliar rat for the LTSDMT day.

### Experiment 3: Is LTSDM stronger after a 4 h interval?

#### Animals

Female rats (n = 20) were obtained from Charles River (St. Constant, Quebec) and were housed in pairs in the Brock University vivarium under standard laboratory conditions on a 12-h light–dark cycle (lights on at 12:00 AM) with free access to food and water. Rats were tested at 140 days of age. Rats were not handled before any experimental treatment other than for regular cage maintenance and to be weighed weekly.

#### Experimental design

The experimental methods were the same as in experiment two with the exception that the SAT was administered once only and the LTSDMT occurred 4 h later.

#### Statistics

All analyses were carried out using SPSS (version 26) and consisted of two and three-way repeated measures analysis of variance (ANOVA) followed by Bonferroni corrected planned comparisons, one sample t-tests, and Pearson correlations [33]. Results are presented as mean ± SEM, and the alpha level of significance was p < 0.05 (two-tailed). Graphs were created using GraphPad Prism (version 8.4.3).

## Results

### Experiment 1: Do both male and female Long Evans rats show evidence of LTSDM when tested after a 24 h interval?

#### Social approach

**Fig 1:**
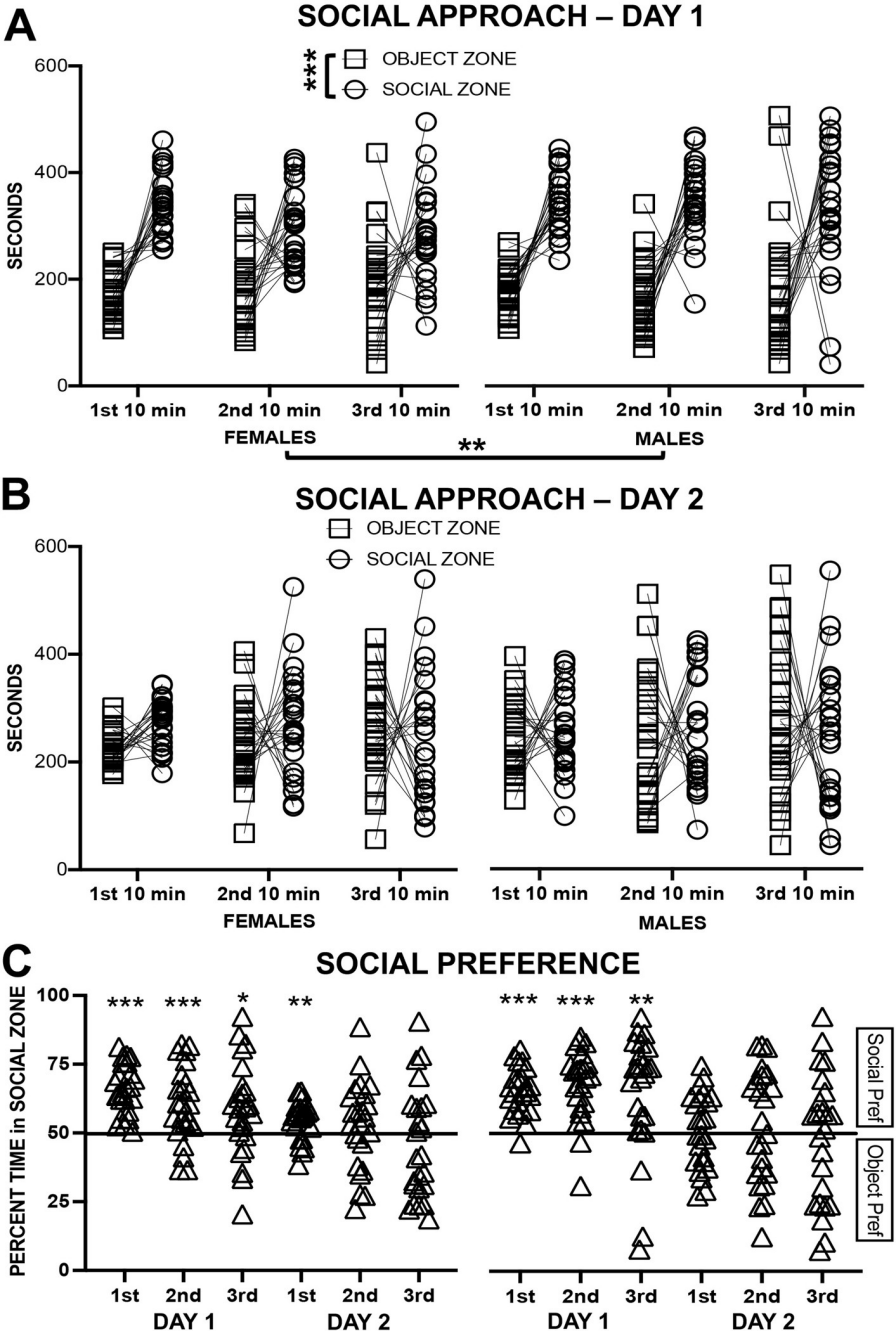
Experiment 1 Social Approach Test (SAT) in female and male rats. (A) Time spent in the object zone and in the social zone across three 10 min blocks of time on the first test day; more time in social zone, *** p < 0.001. (B) Time spent in the object zone or in the social zone across three 10 min blocks of time on the second test day. (C) The percentage of time in the social zone across three 10 min blocks of time on both test days; social preference above chance * p < 0.05; **p < 0.01; *** p < 0.001.

Based on a zone (social vs object) by timepoint (1^st^, 2^nd^, 3^rd^ 10 min) by sex (male vs female) ANOVA for Day 1, rats spent more time in social than in the object zone (F_1,46_ = 71.87, p < 0.001, np^2^ = 0.610) (see Fig 1A). Further, the amount of time in zones (F_1,46_ = 5.76, p = 0.004, np^2^ = 0.111) was higher in the 1^st^ 10 min than in the other 10 min blocks (p = 0.005 and p = 0.012, respectively), and female rats spent more time in zones than did male rats (F_1,46_ = 9.59, p = 0.003, np^2^ = 0.172). On Day 2, no main effect nor interaction was significant (see Fig 1B).

One sample t-tests (two-tailed) were used to determine whether the preference for the social zone was significantly above chance. It was so for all three timepoints on Day 1 and in the 1^st^ 10 min on Day 2 for female rats (p < 0.001, p < 0.001, p = 0.02, and p = 0.004, respectively), and for all three timepoint on Day 1 and for no timepoint on Day 2 for male rats (p < 0.001, p < 0.001, and p = 0.003, respectively) (see Fig 1C).

There was no difference in distance travelled between Day 1 and Day 2 (F_1,46_ = 0.27, p < 0.064, np^2^ = 0.006), and female rats travelled greater distances than did male rats (F_1,46_ = 6.47, p = 0.014, np^2^ = 0.123).

#### Social memory

**Fig 2:**
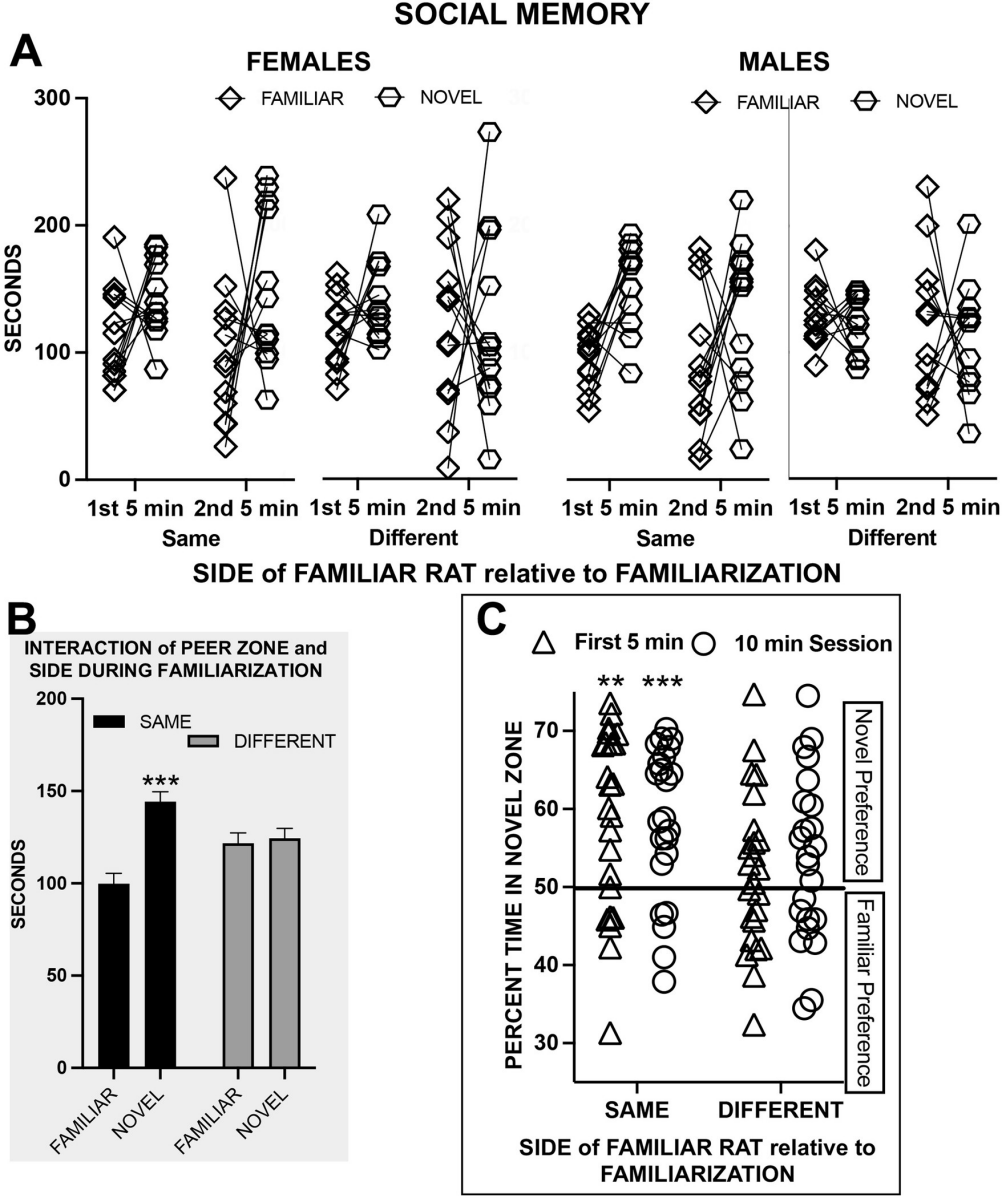
Experiment 1 Long-Term Social Discrimination Memory Test (LTSDMT) in female and male rats. (A) Time spent in the familiar peer zone or in the social peer zone in the first and second 5 minutes of the test when the familiar rat was in the same or different compartment of the test arena. (B) The statistical results for (A) indicating the estimated marginal means (+ s.e.m.) for the interaction of location during familiarization and zone. Time in novel peer zone was higher than in the familiar peer zone only when the familiar rat was in the same zone as during familiarization; *** p < 0.001. (C) The percentage of time in the novel peer zone in the first 5 min and for the 10 min session; novel chance above chance, * p < 0.05, ** p < 0.01.

A peer zone (familiar vs novel) by side of familiar rat relative to familiarization (same vs different) by time block (first 5 min vs second 5 min) by sex (females vs male) ANOVA (see Fig 2A) indicated that rats spent more time in zones in the first than in the second 5 min (F_1,44_ = 6.78, p = 0.013, np^2^ = 0.133) and that female rats spent more time in zones than did male rats (F_1,44_ = 5.49, p = 0.024, np^2^ = 0.111). There was an interaction between peer zone and social side during familiarization (F_1,44_ = 8.48, p = 0.006, np^2^ = 0.197) whereby the higher time in the novel than in the familiar peer zone was significant only when the familiar rat was in the same side as during familiarization (p < 0.001) (see Fig 2B).

One sample t-tests (two-tailed) were used to determine whether each side during familiarization group’s preference for the novel was significantly above chance for the first 5 min block and for the 10 min session. When the familiar rat was on the same side as during familiarization, the preference of rats for the novel was significant for the first 5 min (p = 0.001) and for the 10 min test session (t_23_ = 4.47, p < 0.001). When the familiar rat was on the different side from that during familiarization, the preference was not significant for either the first 5 min (p = 0.372) or the 10 min session (p = 0.058), which suggests that the novelty of the new location of the familiar rat competed against the novelty of a new peer (see Fig 2C).

**Fig 3:**
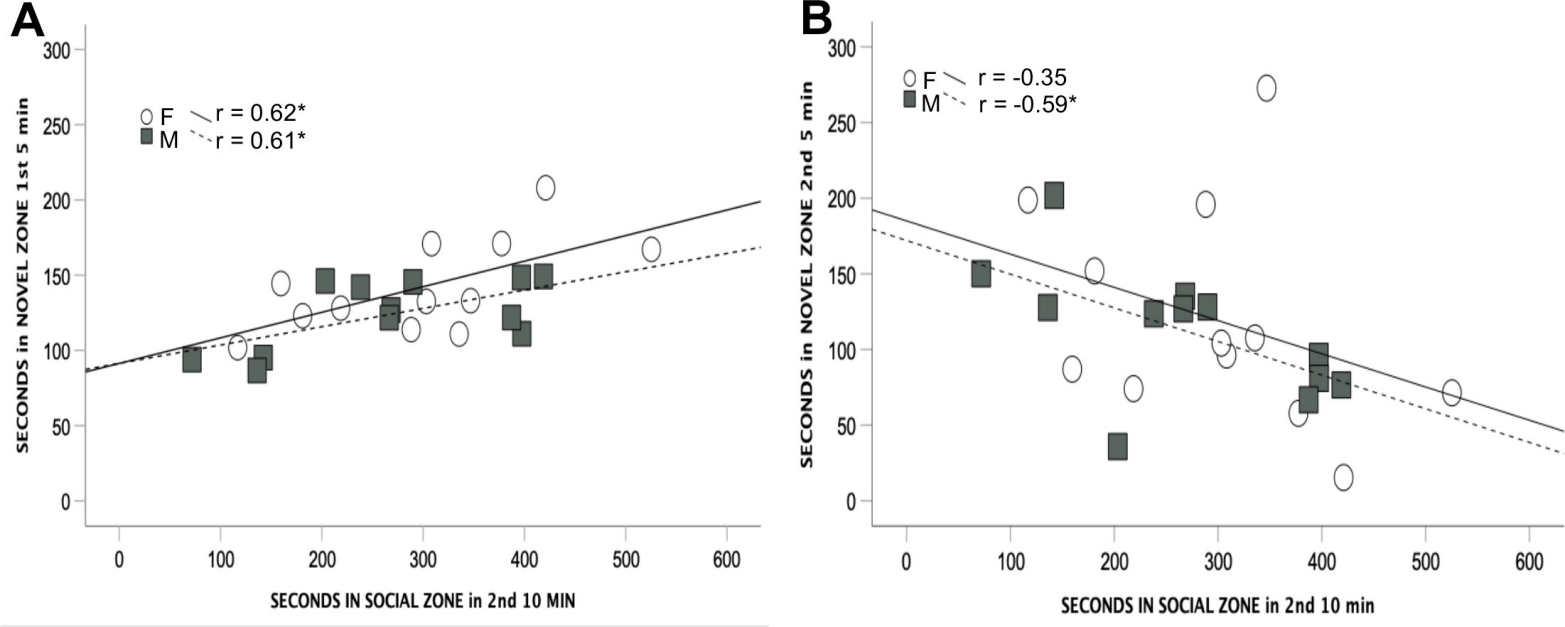
Depiction of correlations from Table 1. Correlation between time spent in the social zone in the 2^nd^ 10 min block of the social approach test and time spent in the novel zone in (A) the 1st 5 min and (B) the 2^nd^ 5 min of the long-term social discrimination memory test. * p < 0.05.

There was no correlation between time spent with the peer during familiarization and preference for the novel peer for either the same side (r_24_ = 0.155) or different side (r_24_ = 0.170) group. The only significant correlations were a positive association between measures in the social approach test for the 2^nd^ min session and time the first 5 min of the LTSDM test that were then negative for the second 5 min of the LTSDM test. See Table 1 for the correlations among all the measures and Fig 3 for scatterplot examples of the positive and negative correlations.

**Table 1:** Correlations between the social approach and the social memory measures.

SAME SIDE								
	Social 30 min	Social 1^st^ 10 min	Social 2^nd^ 10 min	Social 3^rd^ 10 min	Social Pref 30 min	Social Pref 1^st^ 10 min	Social Pref 2^nd^ 10 min	Social Pref 3^rd^ 10 min
Novel time 1^st^ 5 min	-.228	-.365	-.044	.-.190	-.255	-.403	-.051	-.220
Novel time 2^nd^ 5 min	.137	.207	.061	.079	-.028	.127	-.096	-.026
Novel Pref 1^st^ 5 min	-.165	-.322	-.061	-.098	-.168	-.310	-.029	-.122
Novel Pref 10 min	.155	-.005	.207	.092	.019	-.058	.108	-.010
**DIFFERENT SIDE**								
	Social 30 min	Social 1^st^ 10 min	Social 2^nd^ 10 min	Social 3^rd^ 10 min	Social Pref 30 min	Social Pref 1^st^ 10 min	Social Pref 2^nd^ 10 min	Social Pref 3^rd^ 10 min
Novel time 1^st^ 5 min	.269	.109	.622**	.193	.268	.147	.603**	.174
Novel time 2^nd^ 5 min	-.075	-.098	-.415*	-.206	-.035	-.096	-.423*	-.186
Novel Pref 1^st^ 5 min	.283	.109	.555**	.170	.278	.159	.538**	-.150
Novel Pref 10 min	.170	-.034	-.199	-.260	.213	-.031	-.173	-.239

Note: * p < 0.05

** p < 0.01

### Experiment 2: Is LTSDM evident using older adult stimulus and test rats?

#### Social approach

**Fig 4:**
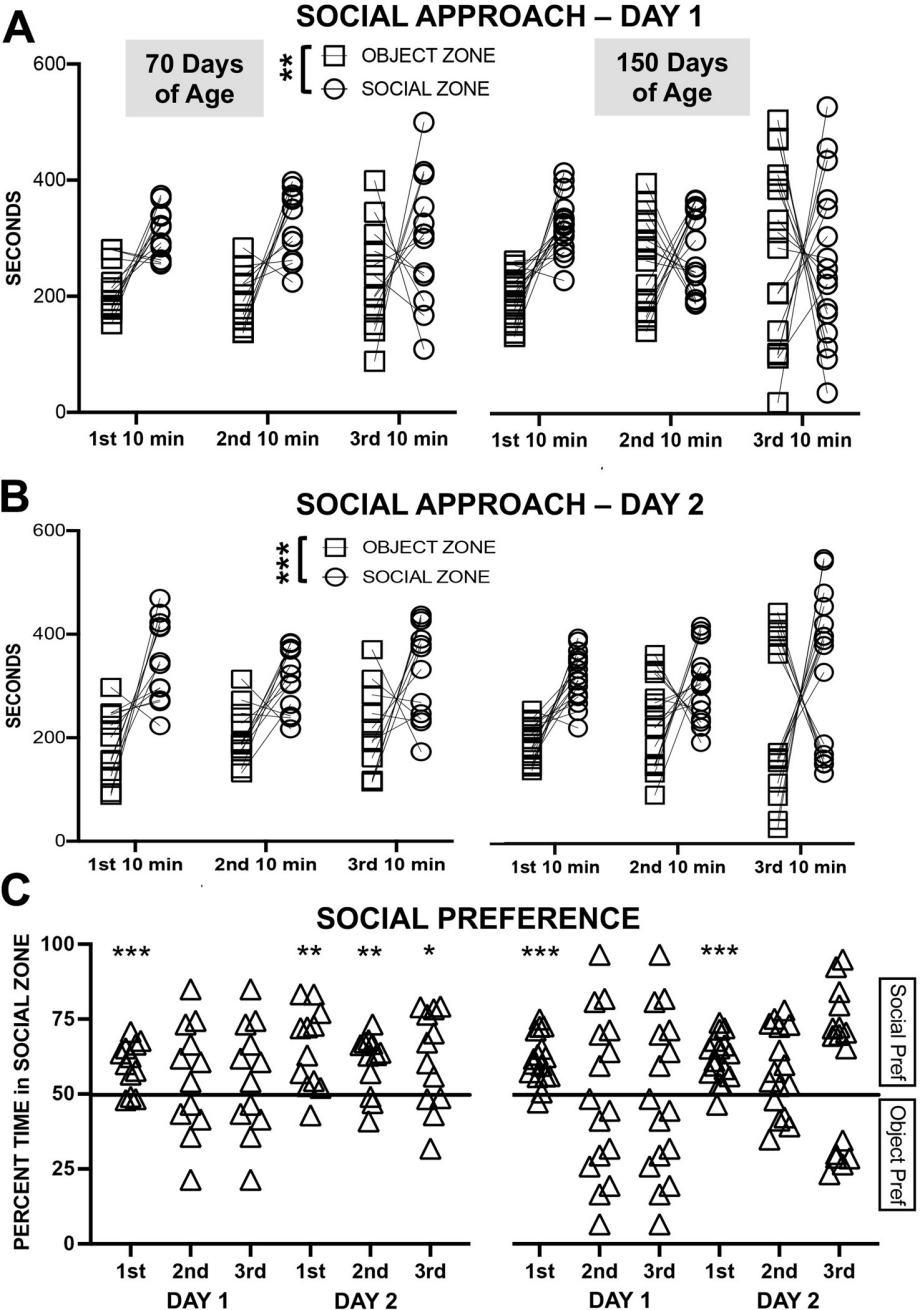
Experiment 2 Social Approach Test (SAT) in female rats either 70 or 150 days of age. (A) Time spent in the object zone or in the social zone across three 10 min blocks of time on the first test day. (B) Time spent in the object zone or in the social zone across three 10 min timepoints on the second test day. (C) The percentage of time in the social zone across three 10 min blocks of time on both test days; social preference above chance, * p < 0.05, ** p < 0.01, *** p < 0.001.

A zone (social vs object) by timepoint (1^st^ 10 min, 2^nd^ 10 min, 3^rd^ 10 min) by age (70 vs 150 days of age) ANOVA indicated that rats spent more time in the social zone than in the object zone on the first test day (F_1,26_ = 8.73, p = 0.007, np^2^ = 0.251; other main effects and interactions all p > 0.07) and on the second test day (F_1,26_ = 21.14, p < 0.001, np^2^ = 0.448) (see Fig 4A and 4B). On the second test day, the interaction of age and timepoint was significant (F_2,52_ = 9.86, p < 0.001, np^2^ = 0.275); rats aged 150 days spent more time in the zones than did rats aged 70 days in the 3^rd^ timepoint (p = 0.006), and rats aged 150 days had an increased time spent in zones in the 2^nd^ (p = 0.012) and 3^rd^ timepoint (p < 0.001) relative to the 1^st^ timepoint and in the 3^rd^ relative to the 2^nd^ timepoint (p = 0.032).

**Fig 5:**
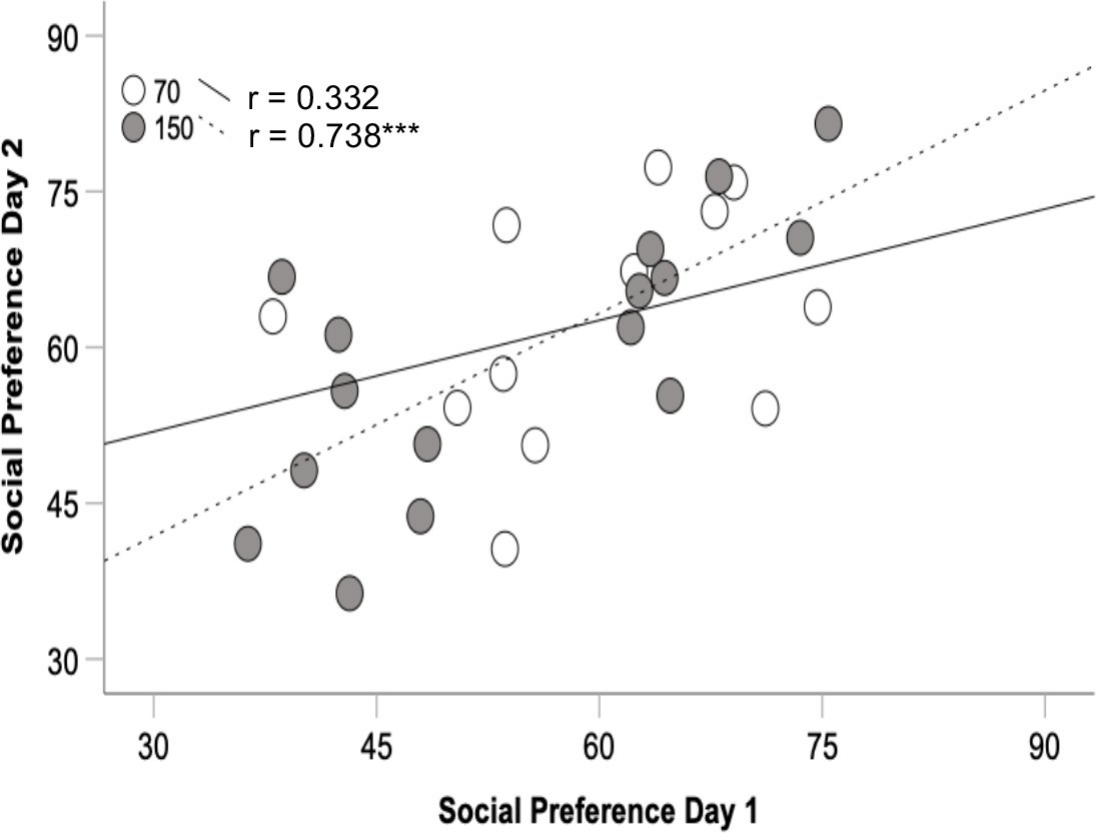
Correlations between the two Social Approach Tests in Experiment 2 in female rats either 70 or 150 days of age. *** p < 0.001.

For rats 70 days of age, the preference for the social zone was significant in the first 10 minutes (p < 0.001) for Day 1, and for all three timepoints for Day 2 (all p < 0.031). For rats 150 days of age, the preference was significant only in the first 10 minutes for both test days (both p < 0.001) (see Fig 4C). Further, the preference for the social zone was correlated significantly between Day 1 and 2 for rats 150 days of age (r_16_ = 0.738, p < 0.001) and not for rats 70 days of age (r_12_ = 0.332, p = 0.292) (see Fig 5). Nevertheless, these two correlations do not differ significantly (z = 1.386, p = 0.19).

There was no effect of age on distance travelled on either test day (all p > 0.05).

#### Social memory

**Fig 6:**
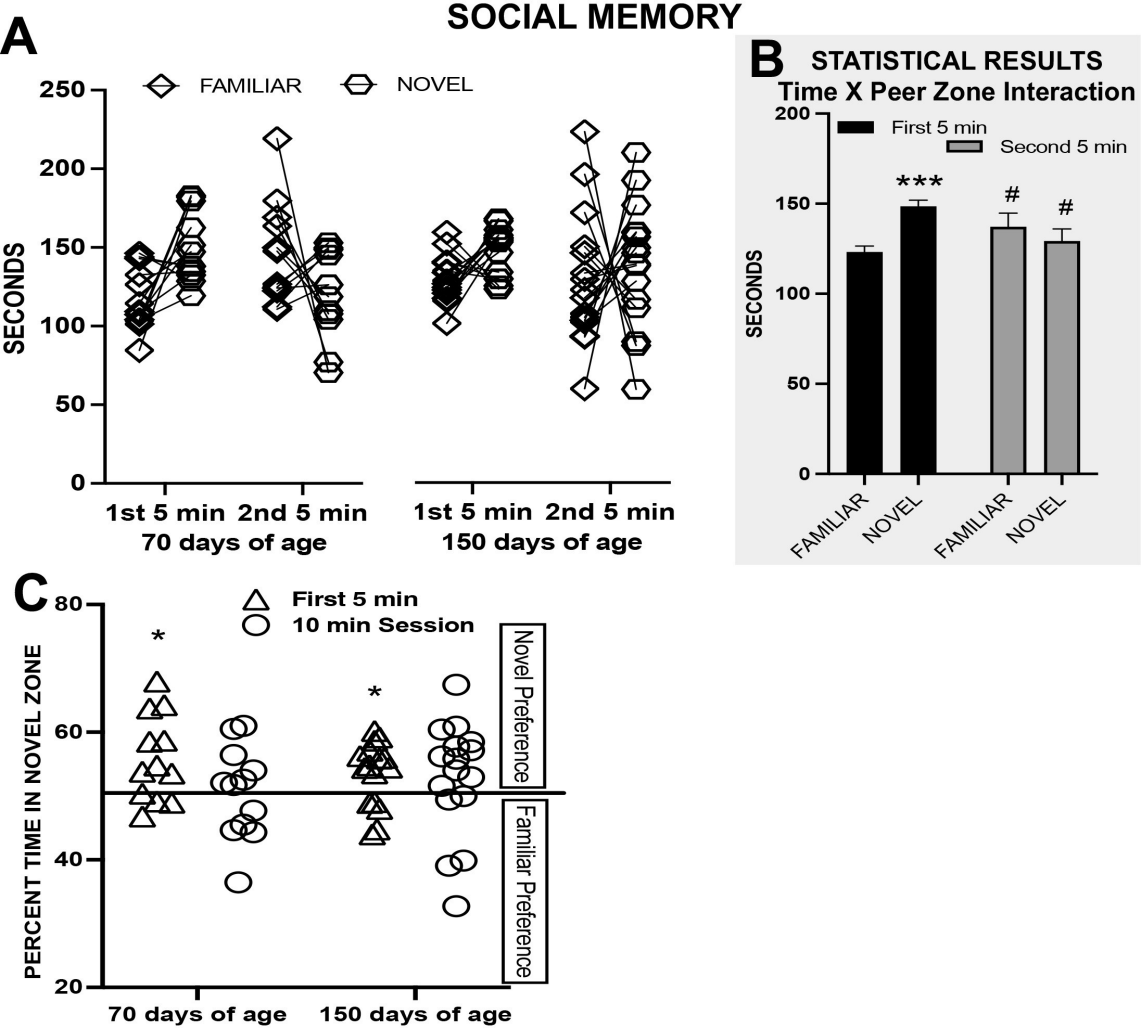
Experiment 2 Long-Term Social Discrimination Memory Test (LTSDMT) in female rats either 70 or 150 days of age. (A) Time spent in the familiar peer zone or in the social peer zone in the first and second 5 minutes of the test when the familiar rat was in the same or different compartment of the test arena. (B) The statistical results for (A) indicating the estimated marginal means (+ s.e.m.) for the interaction of time and peer zone; novel vs familiar difference *** p < 0.001; difference between time points, # p < 0.05. (C) The percentage of time in the novel peer zone in the first 5 min and for the 10 min session; novel preference above chance preference * p < 0.05.

A peer zone (familiar vs novel) by time block (first 5 min vs second 5 min) by age (70 vs 150 days of age) ANOVA revealed an interaction between zone and time block (F_1,26_ = 7.41, p = 0.011, np^2^ = 0.22) whereby rats spent more time in the novel peer zone than in the familiar peer zone in the first 5 min only (p < 0.001) (see Fig 6A and 6B). Time in the novel peer zone decreased from the first to second time block (p = 0.005) and increased in the familiar peer zone (p = 0.038). The main effect of age and interactions with age were not significant (p > 0.05).

The preference for the novel rat was above chance in the first 5 min and not for the 10 min session for both P70 rats (t_11_ = 2.99, p = 0.012 and p = 0.785) and P150 rats (t_15_ = 2.75, p = 0.015 and p = 0.242) (see Fig 6C).

There was no correlation for either age group between the social approach test measures and the social memory measures (all *p* > 0.50, see Table 2).

**Table 2:** Correlations between the social approach and the social memory measures.

70 DAYS OF AGE								
	Social 30 min	Social 1^st^ 10 min	Social 2^nd^ 10 min	Social 3^rd^ 10 min	Social Pref 30 min	Social Pref 1^st^ 10 min	Social Pref 2^nd^ 10 min	Social Pref 3^rd^ 10 min
Novel time 1^st^ 5 min	.155	.228	.375	-.060	.142	.251	.262	-.041
Novel time 2^nd^ 5 min	-.355	-.162	-.211	-.446	-.320	-.137	-.172	-.445
Novel Pref 1^st^ 5 min	.105	.091	.202	.064	.140	.129	.175	.112
Novel Pref 10 min	-.214	-.085	-.072	-.282	-.144	-.047	-.014	-.227
**150 DAYS OF AGE**								
	Social 30 min	Social 1^st^ 10 min	Social 2^nd^ 10 min	Social 3^rd^ 10 min	Social Pref 30 min	Social Pref 1^st^ 10 min	Social Pref 2^nd^ 10 min	Social Pref 3^rd^ 10 min
Novel time 1^st^ 5 min	-.005	.371	.174	-.214	.050	.432	.261	-.179
Novel time 2^nd^ 5 min	-.251	.004	.127	-.428	-.146	.027	.280	-.366
Novel Pref 1^st^ 5 min	.030	.330	.351	-.244	.092	.445	.426	-.211
Novel Pref 10 min	-.170	.116	.250	-.412	-.068	.178	.391	-.353

### Experiment 3: Is LTSDM stronger after a 4 h interval?

#### Social approach

**Fig 7:**
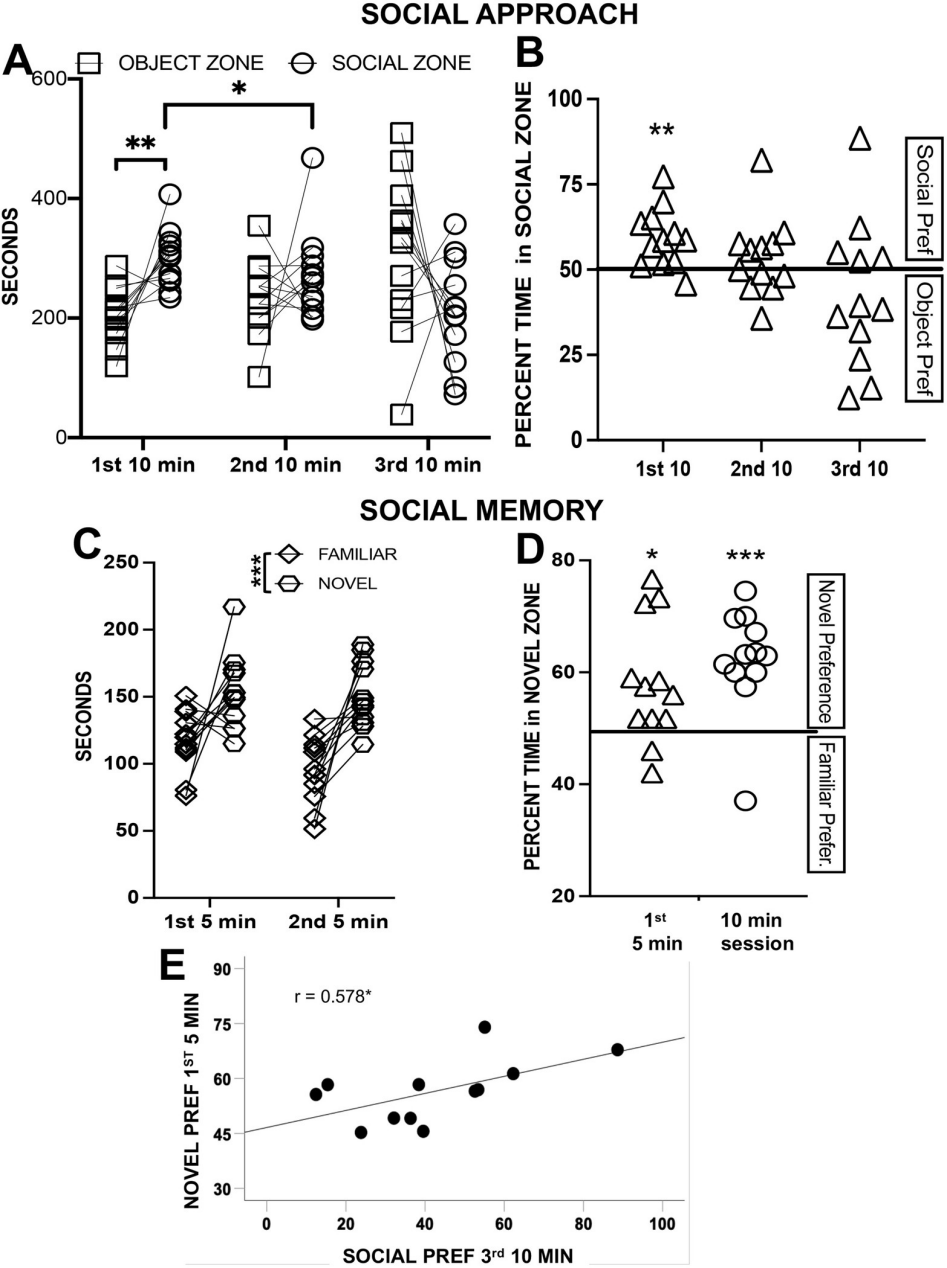
Experiment 3 in female rats. (A) Difference in time spent in the object zone and in the social zone in the Social Approach Test (SAT) is significant only in the first 2 blocks of 10 min *p < 0.05, **p < 0.01. (B) The percentage of time in the social zone; social preference above was chance only in the 1^st^ 10 min *p < 0.05. (C) More time spent in the novel peer zone than in the familiar peer zone (long-term social discrimination memory test; LTSDMT) 4 h after the SAT in female rats, *** p < 0.001. (D) The percentage of time in the novel peer zone was significantly above chance,* p < 0.05, *** p < 0.001. (E) Correlation between social preference in the 3^rd^ 10 min of the SAT and novel preference in the 1^st^ 5 min of the LTSDMT, * p < 0.05.

The interaction of timepoint (1^st^ 10 min, 2^nd^ 10 min, 3^rd^ 10 min) and zone (social, object) was significant (F_2,22_ = 8.52, p = 0.002, np^2^ = 0.436); the time spent in the social zone was higher than time in the object zone in the first 10 min (p = 0.004), and that only the decrease in time in the social zone from the first to second time block was significant (p = 0.028) (other comparisons p > 0.09) (see Fig 7A). The preference for the social zone was significant in the first 10 minutes only (p = 0.004) (see Fig 7B).

#### Social memory

A timepoint (first 5 min, second 5 min) by peer zone (familiar, novel) indicated that rats spent more time with the novel rat than the familiar rat (F_1,11_ = 23.79, p < 0.001; np^2^ = 0.684) and that rats spent more time in the peer zones in the first 5 min than in the second 5 min (F_1,11_ = 13.90, p = 0.003, np^2^ = 0.558) (see Fig 7C). The preference for the novel rat was above chance for both the first 5 min (p = 0.012) and for the 10 min session (p < 0.001) (see Fig 7D). The only significant correlation between performance in the social approach test measures and the social memory measures was between social preference in the 3^rd^ 10 min timepoint and novelty preference in the 1^st^ 5 minutes (r_11_ = 0.578; see Fig 7E), although there were many associations of similar strength to those (e.g., r = 0.400 to r = 0.538, see Table 3).

**Table 3:** Correlations between the social approach and social memory measures.

	Social time 30 min^1^	Social time 1^st^ 10 min^2^	Social time 2^nd^ 10 min^3^	Social time 3^rd^ 10 min^4^	Social Pref 30 min^5^	Social Pref 1^st^ 10 min^6^	Social Pref 2^nd^ 10 min^7^	Social Pref 3^rd^ 10 min^8^
Novel time 1^st^ 5 min	.264	.374	-.020	.257	.360	.401	-.021	.420
Novel time 2^nd^ 5 min	.056	-.027	-.116	.190	.144	.003	-.130	.283
Novel Pref 1^st^ 5 min	.465	.513	.200	.353	.538	.487	.171	**.578** ^*^
Novel Pref 10 min	.257	-.055	.102	.378	.289	-.068	.031	.460

Bold font indicates significance; *p < 0.05

The preference for the novel rat in this experiment was not higher after the 4 h interval than it was after a 24 h interval for rats of either age in Experiment 2 in the first 5 min of session (F_2,37_ = 0.81, p = 0.421, np^2^ = 0.042). For the 10 min session, the preference for the novel rat after a 4 h interval was higher in this experiment (F_2,37_ = 3.77, p = 0.032, np^2^ = 0.169) than after 24 hr when compared to both the 70 (p = 0.012) and 150 (p = 0.045) day old rats in Experiment 2.

## Discussion

The present studies addressed the lack of research on long-term social memory in rats, particularly in female rats and Long Evans rats of either sex [4]. The experiments indicated that long-term social memory can be obtained reliably in a social discrimination procedure 24 hours after a 30-minute familiarization session in Long Evans rats of both sexes. The preference for the novel peer was greater after a 4 hour interval than after a 24 hour interval, which supports the social discrimination test as a memory test, given the expectation of reductions in memory over time [34]. Previous social discrimination tests have involved familiarization sessions of one hour to investigate long-term memory (e.g., [7, 18–22]), although there is one report that a 10-minute familiarization session resulted in long-term social memory [23]). That the familiarization session can be reduced to 30 minutes has many practical advantages, such as increasing the number of individuals tested each day. Further, whereas most research has relied on juvenile rodents as the stimulus, we show that same-sex peers are effective stimuli and are effective over a wider range of ages of adulthood than could be obtained in the juvenile period of life; indeed, 70 and 140 day-old-rats showed evidence of social memory using age-matched controls as the stimuli. In tests of short-term social memory, the characteristics of the stimulus mattered, with evidence of longer memory for adult ovariectomized female than for juvenile male stimulus rats [35]. Whether 30 minutes of familiarization is sufficient for long-term memory with other social stimuli remains to be determined.

We also found that it is essential to keep stimulus rats in the same location across experimental days; no long-term social memory was obtained when the familiar rat was placed in a new location for the discrimination trial (experiment 1). Similarly, the social preference evident in the social approach test (difference in time spent near peer versus near an object) on Day 1 was absent on Day 2 when the location of object and peer was reversed. These results suggest that different novelty cues can affect performance in social behaviour tests. The novelty of a new location of a familiar peer competed with the novelty of a new peer, and the novelty of a social stimulus in a new location and its absence in the opposite location reduced social preference in the social approach test. It is well established that rats will spend more time investigating a novel object than a familiar object, and more time investigating a familiar object in a novel location than a familiar object in the familiar location [2, 3]. These results suggest that social peer novelty does not surpass other forms of novelty in gaining rats’ attention.

No sex difference was evident in social approach or in long-term social memory in experiment 1. Others have reported that female rats have better social memory performance than do males in short-term tests despite less investigation of juveniles than males investigate [36]. The lack of a sex difference may be because estrous cycle was not taken into consideration; in mice, females had higher social memory during proestrous when both estradiol and progesterone are high, and the reduced social memory of ovariectomized females was rescued by administration of estradiol (reviewed in [37]). Memory-enhancing effects of estradiol have been reported for several memory tasks [38, 39]. Female rats, however, travelled greater distances than did male rats in the apparatus; higher levels of activity in female than in male rats is reported commonly [40, 41].

There was no relationship between time spent near the peer during familiarization and the preference for a novel peer (i.e., the measure of social memory). The lack of a relationship may be because many of the cues from the peer during familiarization such as volatile odour molecules and ultrasonic vocalizations are available to the test rat irrespective of its location in the apparatus. Rats, however, are thought to depend on non-volatile olfactory cues for social recognition [12]. Interaction between the test and stimulus rats during familiarization close to the mesh is required to access such cues, and it may be the number of bouts near the mesh during a session is more important than duration at the mesh [4]. Nevertheless, 30 minutes in an apparatus with a peer behind mesh was sufficient to produce long-term social memory. There was variability, however, in whether the social novelty preference would be evident in the first five minutes or only evident when the total 10-minute session. Thus, we recommend the use of a 10-minute test session and both timeframes in tests of long-term social memory.

In the social approach test, more time was spent near the social peer than the object in experiment 1 (although not when the peer was in a new location on Day 2) and in experiment 2, and in the first 10 minutes of the session in Experiment 3. When the percent time in the social zone is considered, the first 10 minutes of the tests showed the most reliable social preference across all three experiments. Our results also indicate that there is much variability within individuals in performance in the social approach test. Although social preference was positively correlated on Day 2 with social preference on Day 1 in experiment 2 when the social stimulus was kept on the same side of the apparatus each day, Day 1 only accounted for 11% of the variance in 70-day-old rats and 54% of the variance in 150-day-old rats in social preference on Day 2. Although greater consistency may be found with shorter social approach tests, the 30-minute session was chosen to serve the purpose of being a familiarization session for the social memory test. Social approach test sessions typically are less than 15 min in length, and thus our results suggest when using the social approach test as a 30-minute familiarization for social memory, only the first 10 minutes should be considered to measure social. Despite some modest correlations, there was no evidence of a robust relationship between time spent in the social zone (near a peer rather than near an object) during familiarization and subsequent social memory. The lack of a relationship may be because many of the cues to the identity of the peer are available to the test rat irrespective of its location in the test arena.

The majority of research on social memory has been conducted in mice [4]. In rats, far more is known about the neural underpinnings of short-term than long-term social memory, and there is evidence that different mechanisms are involved in each type of memory. For example, when targeting glutamatergic signalling in the medial prefrontal cortex after the familiarization session, the consolidation of short-term social memory was attenuated by blockade of glutamatergic NMDA receptors whereas the consolidation of long-term social memory was attenuated by the blockade of AMPA/kainate receptors in male rats [20]. Several other brain regions were identified as important in male rats for long-term social memory for juvenile rats 24 h after a one-hour familiarization session. Antagonism of various neurotransmitter systems or impeding protein synthesis with anisomycin in the CA1 region of the hippocampus or the basolateral nucleus of the amygdala disrupted long-term social memory in male rats [16]. Oxytocin signalling in the medial amygdala [7, 18] and in the paraventricular nucleus and supramammillary nucleus [22] were required for long-term social memory in male rats. The extent to which females rely on the same neural mechanisms remains to be determined. Reducing the time required for familiarization sessions such as in these latter studies will facilitate future investigations.

In conclusion, we have established a social discrimination protocol that is efficient and effective for investigating both social approach and long-term social memory in both sexes, thereby addressing the lack of research in female rats in long-term social discrimination research. We have preliminary evidence that the protocol in experiment 2 is effective in investigating the role of oxytocin signalling in the lateral septum in long-term social memory (Pirri & McCormick, in progress). The lateral septum also is involved in regulating many social behavioural [42], and there is evidence for a role of oxytocin signalling in short-term social memory in rats [35]. Day 1 of the social approach test allows for groups to be matched after stereotaxic surgery before any pharmacological treatment on Day 2 and subsequent testing, although two days of a social approach test is not required for long-term social discrimination memory. This protocol satisfies the goal of an efficient procedure to investigate both the possibility of enhancing or diminishing social approach and social memory with experimental manipulations in both sexes.
